# Thermodynamic versus kinetic basis for the high conformational stability of nanobodies for therapeutic applications

**DOI:** 10.1002/1873-3468.15064

**Published:** 2024-11-26

**Authors:** Atanasio Gómez‐Mulas, Mario Cano‐Muñoz, Eduardo Salido Ruiz, Angel Luis Pey

**Affiliations:** ^1^ Departamento de Química Física Universidad de Granada Spain; ^2^ Center for Rare Diseases (CIBERER) Hospital Universitario de Canarias, Universidad de la Laguna Tenerife Spain; ^3^ Departamento de Química Física, Unidad de Excelencia en Química Aplicada a Biomedicina y Medioambiente e Instituto de Biotecnología Universidad de Granada Spain

**Keywords:** conformational stability, nanobodies, protein unfolding, therapeutics, thermodynamics

## Abstract

Nanobodies (NB) are powerful tools for biotechnological and therapeutic applications. They strongly bind to their targets and are very stable. Early studies showed that NB unfolding is reversible and can be analyzed by equilibrium thermodynamics, whereas more recent studies focused on their kinetic stability in very harsh conditions that are far from storage or physiological temperatures (4–37 °C). Here, we show that the thermodynamic view of NB stability holds in a wide range of temperatures (18–100 °C). The thermodynamic stability of three different NBs did not correlate with binding affinity for their target. Alpha‐Fold 2 analyses of these NBs showed structural differences in the binding site and hydrogen bond networks. We expect that our approach will be helpful to improve our capacity to enhance structure–function–stability relationships of NB.

## Graphical abstract

Nanobodies (NB) are attractive tools for biotechnological and therapeutic applications. NB have high stability, but their denaturation mechanism is unclear. We show that three NB with therapeutic potential and structural similarity largely differ in their thermodynamic stability in a wide range of temperatures. We also show that the NB stability does not correlate with the binding affinity for the target. Our work may help design more stable NB.

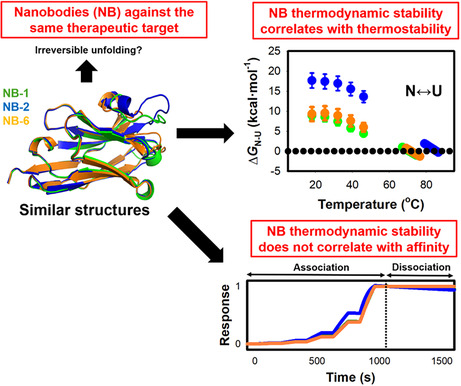

## Abbreviations

**AGT‐LM**, minor allele of the human alanine glyoxylate aminotransferase

**BB**, binding buffer

**CDR**, complementarity‐determining region

***C***_**m**_, concentration of GdmHCl for half‐unfolding

***C***_**p,app**_, unfolding apparent heat capacity

**DSC**, differential scanning calorimetry

**GdmHCl**, guanidinium hydrochloride

**HEPES**, *N*‐(2‐hydroxyethyl)piperazine‐*N*′‐(2‐ethanesulfonic acid)

**HPLC/ESI‐MS**, high‐performance liquid chromatography coupled to electrospray ionization mass spectrometry

**HVL**, hypervariable loop

**I**, fluorescence emission intensity

**IMAC**, immobilized metal affinity chromatography

**IPTG**, isopropyl β‐d‐1‐thiogalactopyranoside

***K***, unfolding equilibrium constant

***k***_**agg**_, aggregation rate constant

**ku**, unfolding rate constant

**LB**, Luria‐Bertani

**LBK**, LB with kanamycin

***m***_**eq**_, chemical unfolding cooperativity

**NB**, nanobody

**NB‐AGT‐1, ‐2 and ‐6**, NB‐1, ‐2 and ‐6 raised against AGT‐LM

**SDS/PAGE**, polyacrylamide gel electrophoresis in the presence of sodium dodecyl sulphate

***T***_**m**_, temperature at which *K* = 1

**Δ*C***_**p**_, unfolding heat capacity change

**Δ*G***, unfolding free energy change

**Δ*H***_**cal**_, unfolding calorimetric change in enthalpy

**Δ*H***_**VH**_, unfolding Van't Hoff enthalpy

Protein stability is a key factor for therapeutic application of proteins since therapeutic proteins must retain their activity at *in vivo* conditions [1, 2, 3]. However, the relationships between *protein stability* (thermodynamic and kinetic stability, local unfolding…) and the shelf life of therapeutic proteins is not well understood. Here we address how the thermal stability of three nanobodies (NB) raised to correct protein misfolding in a rare genetic disease (primary hyperoxaluria type 1, [4, 5, 6]) correlate with thermodynamic stability in a wide range of temperatures.

Nanobodies are single‐domain and antigen‐specific fragments derived from the heavy‐chain antibodies of camelids, such as alpacas, llamas, and dromedaries, with a great potential as therapeutic agents [7, 8, 9, 10, 11, 12, 13]. NB bind to their targets through three complementarity‐determining regions or CDR (CDR1, CDR2, and CDR3) that contain each a hypervariable loop (HVL 1–3) [7]. These CDR (particularly CDR3) determine their high specificity and affinity for its target [7]. In an early study, it was described that NB unfolding is highly reversible at low temperatures and that NB display high conformational stability, with an unfolding free energy in the range of 10–15 kcal·mol^−1^ and resembling well a two‐state equilibrium unfolding model [14]. Thus, considering simple Lumry‐Eyring models, one could consider that the shelf life of NBs is associated with the thermodynamics (and kinetics?) of unfolding under storage (e.g. 4 °C) or working (e.g. 37 °C) temperatures [15, 16, 17]. As expected, incubation of NB at very high temperatures [in the range 70–85 °C, close to the apparent melting temperature (*T*_m_) of the NB] led to irreversible denaturation, and this approach was alternatively used to explain the different thermostability of different NB [18, 19]. However, we must note that studies carried out at such high temperatures imply rather long (kinetic) extrapolations to low temperatures, and that the irreversible processes dominating at high temperatures may not hold at low temperatures [i.e. different irreversible processes may show different activation energies (*E*_a_) according to a simple Arrhenius analysis].

Protein stability analyses derived from thermal denaturation experiments are not always easy to be carried out strictly and difficult to understand deeply [20]. Two key factors necessary to apply a suitable model to yield thermodynamic and/or kinetic properties from protein thermal denaturation scans are the experimental reversibility and the scan rate dependence [20]. A high reversibility of the thermal transition(s) has been traditionally taken as a *bona fide* proof for applying thermodynamic analysis to thermal denaturation experiments, although the scan rate dependence must be also taken into account [20, 21]. For instance, thermal denaturation of human phenylalanine hydroxylase has been shown to be irreversible but its scan rate dependence vanishes at moderate scan rates (about 1 K·min^−1^) allowing partial characterization of its unfolding thermodynamics using equilibrium models as long as the post‐transition behavior (highly distorted by irreversible processes) is not taken into account [22]. Importantly, early works carried out with a wide variety of NB showed that their reversibility upon thermal denaturation is moderate‐high (typically 50–100%) when moderate scan rates are used (0.5–1 K·min^−1^) [14, 23, 24], whereas other authors have described thermal denaturation of NB as a kinetically controlled process and not dictated by equilibrium thermodynamics [17, 18]. Therefore, it is not clear how the high thermodynamic stability of NB at room (or storage or physiological) temperature is connected (or not) with a high thermal stability and which are the structural‐energetic basis of their stability (thermodynamic and/or kinetic stability, local and global stability).

In this work, we have revisited the relationships between equilibrium and thermal stability of different NB and confirmed that their thermostability can be explained well from a higher thermodynamic stability at all temperatures, particularly at working conditions (around 37 °C for a therapeutic NB). The binding affinity of these three NB (named NB‐AGT‐1, ‐2 and ‐6) vary in three orders of magnitude, *K*_d_ ranging from 3.8 (NB‐AGT‐1) to ~ 5 × 10^−3^ (NB‐AGT‐6) nm to AGT‐LM (the *minor* allele of alanine:glyoxylate aminotransferase associated with the rare disease name primary hyperoxaluria type I, OMIM # 259900) [25] (Fig. S1). We combined chemical and thermal denaturation experiments to generate thermodynamic stability curves for these NB that differ in 15 °C in the thermal denaturation temperatures and about 10 kcal·mol^−1^ in the unfolding free energy at room temperature.

## Materials and methods

### Nanobody generation

Nanobody generation was carried out by NabGen Technologies (Marseille, France), conforming to appropriate animal ethics regulations and guidelines. Briefly, a llama was immunized with 500 μg of the purified AGT‐LM protein dimer (purified as described in Ref. [26]). After three injections at 21 days intervals, a blood sample was collected after 54 days and pre‐immune and immune sera were collected.

AGT‐LM was adsorbed on a maxisorp plate (Fisher Scientific, Illkirch Cedex, France). The immune response was analyzed by ELISA using goat anti‐llama‐HRP conjugate (Waltham, MA, USA) and recorded in 96‐well plates at 450 nm on a Safire 2 microplate reader (Tecan, Lyon, France).

A library from blood collected on day 54 upon extraction of mRNA using the RNeasy Mini Kit Qiagen (Les Ulis, France). cDNA was synthesized using the SuperScript IV First strand Synthesis System invitrogen (Fisher Scientific). From this cDNA, the nanobody encoding reading frames were amplified by PCR and then cloned into a phage display vector with a Human influenza hemagglutinin (HA) tag. Enrichment for target‐specific antibodies against AGT was performed using the phage display technique with M13KO7 Helper phage (New England Biolabs, Courcouronnes, France). To immobilize the targets for selection by phage display, an specific adsorption onto the solid surface of a maxisorp plate was used.

Following two rounds of panning, 12 clones from round 1 and 12 clones from round 2 were picked and expressed in *Escherichia coli*. Those clones were tested against AGT‐LM with an ELISA using monoclonal anti‐HA antibody produced in mouse (Sigma‐Aldrich, Lezennes, France) as a primary antibody and goat anti‐mouse IgG/IgM HRP conjugated antibody (Millipore, Molsheim, France). Absorbances of wells were recorded at 450 nm on a Safire 2 microplate reader. Twelve positive clones were sent for sequencing and six were selected for biophysical characterized based on their different CDR3 sequences (NB‐AGT‐1 to ‐6). Three of them were analyzed in this work due to their different thermal stabilities.

### NB expression and purification

*Escherichia coli* BL21 (DE3) cells were transformed with the pET‐24(+) vector containing the cDNA of each NB (NB‐AGT‐1, ‐2 and ‐6) and carrying a C‐terminal 6His‐tag. A preculture of 240 mL of LB medium containing 30 μg·mL^−1^ kanamycin (LBK) was inoculated with transformed cells and grown for 16 h at 37 °C. These cultures were diluted into 4.8 L of LBK, grown at 37 °C for 3 h and NB expression was induced by the addition of 0.5 mm IPTG (isopropyl β‐d‐1‐thiogalactopyranoside) and lasted for 6 h at 25 °C. Cells were harvested by centrifugation and frozen at −80 °C for 16 h. Cells were resuspended in binding buffer, BB (20 mm Na‐phosphate, 300 mm NaCl, 50 mm imidazole, pH 7.4) plus 1 mm PMSF (phenylmethylsulfonyl fluoride; Sigma‐Aldrich, Madrid, Spain) and sonicated in an ice bath. These extracts were centrifuged (20 000 ***g***, 30 min, 4 °C) and the supernatants were loaded into IMAC (immobilized metal affinity chromatography) columns (Cytiva, Barcelona, Spain), washed with 40 bed volumes of BB and eluted in BB containing a final imidazole concentration of 500 mm. These eluates were immediately buffer exchanged using PD‐10 columns (Cytiva, Barcelona, Spain) to 50 mm HEPES [*N*‐(2‐hydroxyethyl)piperazine‐N′‐(2‐ethanesulfonic acid); Sigma‐Aldrich]‐KOH pH 7.4, analyzed by SDS/PAGE (Polyacrylamide gel electrophoresis in the presence of sodium dodecyl sulphate) and stored at −80 °C upon flash‐freezing in N_2_. NB‐AGT samples were further purified by loading them onto a SuperDex 75 16/60 size exclusion chromatography column (Cytiva, Barcelona, Spain) using 20 mm HEPES‐NaOH, 200 mm NaCl pH 7.4 as mobile phase at 1 mL·min^−1^ flow rate. Fractions containing NB‐AGTs were collected, concentrated, buffer exchanged to 50 mm K‐phosphate pH 7.4 and stored at −80 °C after flash‐freezing in liquid N_2_. Purity and molecular weight was analyzed again by SDS/PAGE and further checked by high‐performance liquid chromatography coupled to electrospray ionization mass spectrometry (HPLC/ESI‐MS) by the High‐resolution mass spectrometry unit, Centro de Instrumentación Científica (University of Granada) in a WATERS LCT Premier XE instrument equipped with a time‐of‐flight (TOF) analyzer. NB‐AGT concentration was measured using the following molar extinction coefficients (ε_280_) according to their primary sequence: NB‐AGT‐1.‐ 25565; NB‐AGT‐2.‐ 33015; NB‐AGT‐6.‐ 20065, all in m ^−1^·cm^−1^.

### Differential scanning calorimetry

**Fig. 1:**
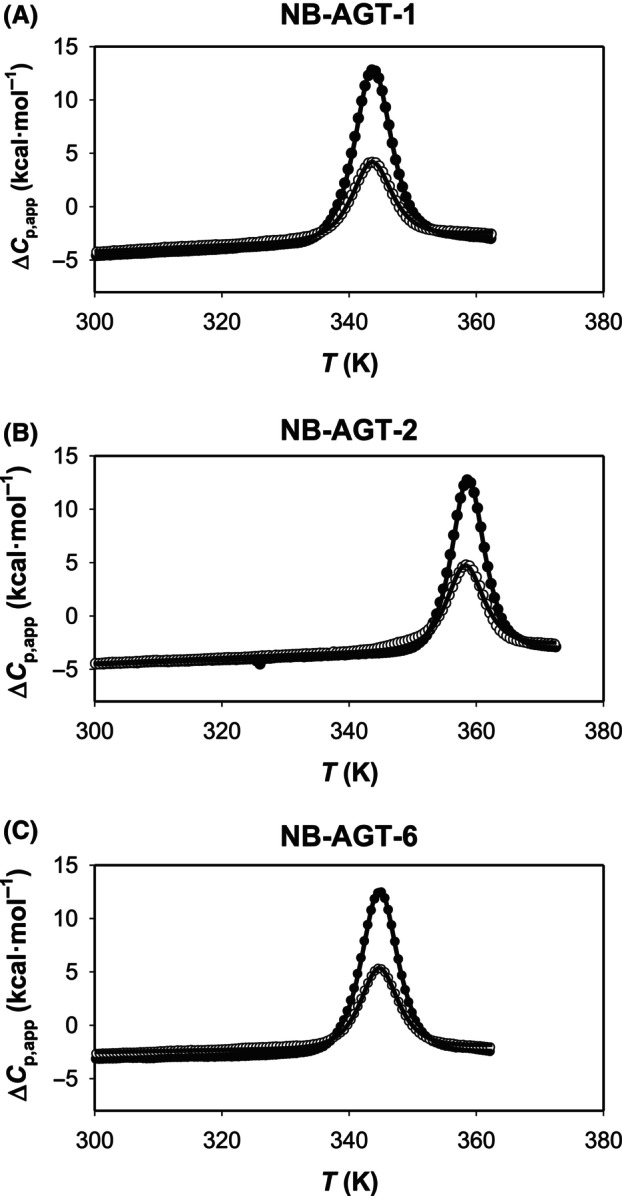
A two‐state equilibrium thermal denaturation model provides a good description of the thermal unfolding of NB‐AGT‐1 (A), NB‐AGT‐2 (B), and NB‐AGT‐6 (C). Closed circles show the upscans and the open circles the rescans. Lines are best‐fit to Eqn (1). Data are from one experiment at a scan rate of 2 K·min^−1^. Thermodynamic denaturation parameters are compiled in Table 1.

Differential scanning calorimetry (DSC) experiments were carried out using a VP‐DSC differential scanning microcalorimeter (Malvern Pananalytical, Malvern, UK) with a cell volume of 137 μL and automated sampling. Experiments were performed in 50 mm K‐phosphate pH 7.4 using 20 μm of NB‐AGT and typically at scan rate of 2 °C·min^−1^ (even though other rates were tested to check for scan rate dependence). Scans were carried out in a temperature range of 20–90 °C (NB‐AGT‐1 and NB‐AGT‐6) or 20–100 °C (NB‐AGT‐2) to allow complete unfolding and to minimize distorsions from irreversible thermal denaturation. Actually, the reversibility is quite significant although not complete, thus supporting the applicability of equilibrium denaturation to determine some relevant unfolding parameters (reversibility was 47 ± 1% for NB‐AGT‐1, 52 ± 2 for NB‐AGT‐2 and 50 ± 1% for NB‐AGT‐6; from calorimetric enthalpies of upscans and rescans shown in Fig. 1). In some DSC experiments, a guanidium hydrochloride (GdmHCl; Sigma‐Aldrich) solution was prepared in 50 mm K‐phosphate pH 7.4 by weight at ~ 6 m in and used at a final concentration of 0–0.9 m (all GdmHCl concentrations were checked by refractive index measurements).

As a first approach to quantitatively evaluate the thermal denaturation behavior of NB‐AGT, we applied a model to analyze the DSC scans in which equilibrium denaturation is not assumed to be a two‐state process (i.e. not only the native and unfolded may be populated). To this end, we used a function in which the temperature (*T* in K) dependence of the apparent heat capacity (*C*_p,app_) was described by Eqn (1):

(1) $$C_{p,\mathrm{app}}\left(T\right)=C_{p,\mathrm{app},N}\left(T\right)+\frac{\Delta H_{\mathrm{cal}}\cdot \Delta H_{\mathrm{VH}}}{R\cdot T^{2}}\cdot \frac{K}{{\left(1+K\right)}^{2}}$$

where *C*_p,app,N_ is the temperature‐dependent apparent heat capacity of the native state (described by a straight line equal to *a* + *b*·*T*), the area under the calorimetric peak or calorimetric enthalpy (Δ*H*_cal_), the Van't Hoff enthalpy (Δ*H*_VH_) that describes the temperature‐dependence of the denaturation equilibrium constant *K* (Eqn 2; the *T*_m_ is the temperature at which *K* = 1) and *R* is the ideal gas constant (1.987 cal·mol^−1^·K^−1^).

(2) $$K=\exp^{\frac{-\Delta H_{\mathrm{VH}}}{R}\cdot \left(\frac{1}{T}-\frac{1}{T_{m}}\right)}$$

**Fig. 2:**
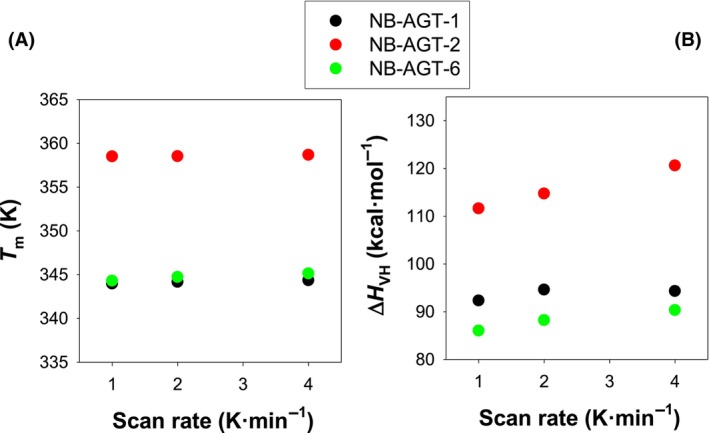
A two‐state equilibrium thermal denaturation model is supported by the weak scan rate dependence of the thermal unfolding of NB‐AGT‐1, NB‐AGT‐2, and NB‐AGT‐6. (A) *T*_m_ for each NB‐AGT determined using a two‐state reversible unfolding model at different scan rates (Eqn 1). (B) The effects of scan rates on the Van't Hoff enthalpies (which describe the dependence of unfolding equilibrium constant on temperature). The errors from fittings cannot be seen since these are smaller than the symbols. Data are from a single experiment at each scan rate.

The unfolding free energy (Δ*G*) at a given temperature was calculated using the well‐known equation Δ*G* = −*R*·*T*·ln *K* at temperatures within the thermal transition. We did not attempt to calculate the denaturation heat capacity (Δ*C*_p_) from DSC scans since the unfolded heat capacity might be significantly affected by the lack of complete reversibility (higher temperatures may largely enhance irreversible processes). Instead, we estimated an average Δ*C*_p_ for the three NB‐AGT from the *T*_m_‐dependent values of Δ*H*_cal_ in the absence or presence of GdmHCl (Fig. 2). Please note that for fitting and display purposes, Temperature is shown as K or as °C along the manuscript.

### Isothermal denaturation by GdmHCl

GdmHCl denaturation of NB‐AGT was carried out by mixing protein solutions with different concentrations of GdmHCl (typically in the 0–6 m range, the actual concentration determined using refractive index measurements). Experiments were carried out in 50 mm K‐phosphate pH 7.4 using 5 μm of NB‐AGTs. Samples were incubated at 4 °C for 24 h and then these were stepwise incubated for 20 min at 18 °C, 25 °C, 32 °C, 39 °C and 46 °C. Once samples equilibrated, protein unfolding was monitored by fluorescence spectroscopy at that temperature, using an excitation wavelength of 295 nm and emission in the range 320–380 nm (both with 5 nm slits). Fluorescence measurements were carried out using 3 × 3 mm quartz cuvettes (Hellma Analytics, LineaLab, Badalona, Spain) in a Cary Eclipse spectrofluorimeter (Agilent Technologies, Madrid, Spain). Blanks in the absence of protein were routinely measured and subtracted. To monitor denaturation, we used two different spectral properties: the ratio of emission intensities at 365 and 335 nm (*I*_365_/*I*_335_) and the spectral center of mass (SCM, in nm, in the 320–380 nm range) the latter calculated using Eqn (3):

(3) $$\mathrm{SCM}\;\left(\mathrm{nm}\right)=\frac{\sum_{\lambda =320}^{\lambda =380}I_{\lambda }\cdot \lambda }{\sum_{\lambda =320}^{\lambda =380}I_{\lambda }}$$

where λ is the emission wavelength and *I*_λ_ is the emission intensity at that wavelength. To extract equilibrium denaturation parameters, we used a two‐state unfolding equilibrium model [27] (Eqn 4):

(4) $$S=\left[S_{N}+m_{N}\cdot \text{[GdmHCl]}+\left(S_{U}+m_{U}\right)\cdot \left(\exp^{\frac{m_{\mathrm{eq}}\cdot \left(\text{[GdmHCl]}-C_{m}\right)}{R\cdot T}}\right)\right]/\;\left[1+\left(\exp^{\frac{m_{\mathrm{eq}}\cdot \left(\text{[GdmHCl]}-C_{m}\right)}{R\cdot T}}\right)\right]$$

where *S* is the experimental spectral feature as a function of the [GdmHCl], *S*_N_ and *S*_U_ are the fitted spectral features for the native and unfolded state baselines at 0 m GdmHCl, respectively, *m*_N_ and *m*_U_ are the slopes of the native and unfolded state baselines, *m*_eq_ describes the unfolding cooperativity, *C*_m_ is the half‐denaturation GdmHCl concentration, *R* is the ideal gas constant and *T* is the experimental temperature (in K). This model provides an excellent description of chemical denaturation of NB‐AGT (Fig. 4) as well as other NB [14]. To yield more accurate *m*_eq_ and *C*_m_ values, data for a given NB‐AGT and temperature using both spectral features were simultaneously (globally) fitted. The product of *C*_*m*_ and *m*_eq_ provides the Δ*G* at a given temperature from GdmHCl‐induced unfolding profiles. A high reversibility for the chemical unfolding of the different NB‐AGT variants monitored by fluorescence was confirmed at different temperatures and GdmHCl concentrations (Fig. S2).

### Stability curves

To carry out simultaneous analyses of denaturation at low temperatures (from GdmHCl denaturation profiles) and high temperatures (in the absence of denaturation) we use an expression for the Gibbs‐Helmholtz integrated equation for a two‐state folder (Eqn 5) [28]:

(5) $$\Delta G\left(T\right)=\Delta H-T\cdot \frac{\Delta H}{T_{m}}+\Delta C_{p}\cdot \left[T-T_{m}-T\cdot \ln\left(\frac{T}{T_{m}}\right)\right]$$

where Δ*G*(*T*) is the temperature‐dependent unfolding free Gibbs Energy, *T* is the experimental temperature, Δ*H*_cal_ is the calorimetric unfolding enthalpy change at the *T*_m_, *T*_m_ is the temperature at which the Δ*G* = 0, Δ*C*_p_ is the change in heat capacity between the folded and unfolded states. Δ*G*(*T*) is calculated from DSC scans using a version of the Van't Hoff equation (Eqn 2). Good fits were obtained for all NB‐AGT by fixing only the *T*_m_ values (Table 1) and allowing Δ*H* and Δ*C*_p_ values to float. Fixing Δ*H* values to those experimentally determined did not significantly improve the fittings or the estimation of Δ*C*_p_ values.

**Table 1:** Equilibrium thermal denaturation parameters for the NB‐AGT as determined by DSC. Parameters were determined using Eqn (1, 2), that does not implicitly assume a two‐state unfolding. The scan rate used was 2 K·min^−1^ (see Fig. 1).

NB‐AGT	Equilibrium thermal unfolding parameters					
	*T*_m_ (°C)	*T*_m_ (K)	Δ*H*_cal_ (kcal·mol^−1^)	Δ*H*_VH_ (kcal·mol^−1^)	Δ*H*_cal_/Δ*H*_VH_	Reversibility
1	70.65 ± 0.01	343.80 ± 0.01	83.3 ± 0.2	92.3 ± 0.3	0.90 ± 0.01	47 ± 1%
2	85.42 ± 0.02	358.57 ± 0.02	87.4 ± 0.2	105.9 ± 0.3	0.82 ± 0.01	52 ± 2%
6	71.68 ± 0.01	344.83 ± 0.01	77.1 ± 0.2	94.1 ± 0.3	0.82 ± 0.01	50 ± 1%

### Binding of affinity of NB to AGT‐LM by surface plasmon resonance (SPR)

Binding affinity for the interaction between NB‐AGT and AGT‐LM was evaluated using a Biacore T200 Surface Plasmon Resonance instrument (Cytiva, Marlborough, MA, USA). NB‐AGT variants were individually and covalently immobilized on NTA chips aiming for greater than 100 response units. To capture the His‐tagged NB‐AGT, the nitrilotriacetic acid (NTA) chip was loaded using a NiCl_2_ 0.5 m solution and 100–200 nm nanobody samples in a HBS‐P+ buffer (10 mm HEPES, 150 mm NaCl, 0.05% V/V Tween 20) passed under a flow rate of 5 μL·min^−1^. The immobilization procedure required activation of the NTA matrix with the same 0.5 m NiCl_2_ solution and activation of the carboxyl groups of the same matrix with a 1 : 1 mixture of *N*‐ethyl‐*N*‐(3‐diethylamino‐propyl)‐ carbodiimide (EDC) and *N*‐hydroxysuccinimide (NHS). Nanobodies followed for the time previously determined under a 5 μL·min^−1^ flow and ethanolamine blocked the remaining activated carboxyl groups, ending the immobilization step. Each NTA chip has four flow cells in which, the first was activated with the EDC/NHS mixture and then blocked with ethanolamine without ligand ever flowing through so as to function as a reference for the others. Solutions used to attach the NB‐AGT to the chip were from Cytiva (Marlborough, MA, USA). Experiments were carried out at 25 °C.

Affinity constants (*K*_a_; their inverse are the dissociation constants, *K*_d_) were determined by performing Sigle Cycle Kinetics (SCK) assays. Serial dilutions of the different AGT variants in HBS‐EP+ (10 mm HEPES, 150 mm NaCl, 3 mm EDTA, 0.05% V/V Tween 20), with concentrations ranging 0.74–60 nm, were injected in 120 s pulses under a 70 μL·min^−1^ flow rate. Resulting sensograms were fitted to a 1 : 1 interaction model using the Biacore T200 Evaluation Software (Cytiva).

### Structural models

To generate structural models of the three nanobodies we used Alpha‐Fold 2 [29] (https://colab.research.google.com/github/sokrypton/ColabFold/blob/main/AlphaFold2.ipynb), using the amino acid sequence of our NB‐AGT and the structure with PDB code 4ZG1 as template as it represents a camelid single‐domain antibody and shares structural similarity with our nanobodies, particularly in the arrangement of the β‐sheet framework and complementarity‐determining regions (CDRs). In this modeling, we used default parameters and the top ranked models were studied for each NB‐AGT (the corresponding PDB files can be found in Data S1). Accessible surface areas in these models were calculated using getarea (https://curie.utmb.edu/getarea.html) [30]. Multiple sequence alignment was carried out using clustal omega (https://www.ebi.ac.uk/jdispatcher/msa/clustalo).

## Results and Discussion

### Global unfolding of NB‐AGT by temperature follows a two‐state equilibrium model

**Fig. 3:**
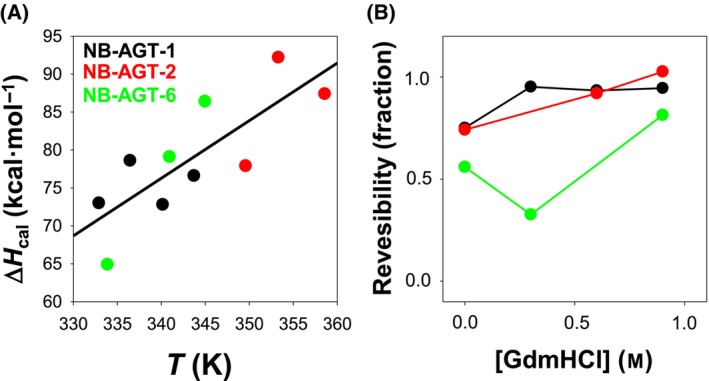
Thermal denaturation of NB‐AGTs with low concentrations of GdmHCl. (A) Calorimetric enthalpy (Δ*H*_cal_) as a function of the *T*_m_. The straight line is an overall fit that provides an unfolding change in heat capacity of 0.80 ± 0.21 kcal·mol^−1^·K^−1^. The Δ*C*_p_ values predicted for proteins of this size are of ~ 1.8 kcal·mol^−1^·K^−1^ [31]. (B) Reversibility (as calculated from calorimetric enthalpies of upscans and rescans) of the thermal denaturation NB‐AGT as function of denaturant concentration. Note that with 0.9 m of denaturant, reversibility is almost complete. Symbols correspond to NB‐AGT‐1 (black), NB‐AGT‐2 (red) and NB‐AGT‐6 (green).

We first carried out thermal denaturation assays by DSC, and checked for their reversibility to focus on differences in thermodynamic stability related to changes in thermostability (rather than investigating aggregation propensities at very high, non‐physiological and non‐pharmaceutically related temperatures, that often required undesired long Temperature‐extrapolations) [18, 19]. As we show in Fig. 1, the reversibility of the thermal transitions for NB‐AGT‐1, ‐2 and ‐6 is about 50%, consistent with previous studies showing that NB thermal unfolding is typically of ~ 50–100% [14, 24]. Additionally, the high symmetry of the peaks and the excellent fits to a simple two‐state denaturation model, support that any source of irreversibility hardly affects the experimental scans and an appropriate analysis using equilibrium thermodynamics can be performed. Furthermore, the marginal effects of the scan rate on the thermodynamic parameters derived from the analysis using this reversible model (Fig. 2 and Table S1) additionally support our DSC analysis. We considered an exception the determination of the unfolded heat capacity that would require heating up the samples to higher temperatures and would likely be affected more severely by irreversible processes [22]. Thus, we did not include the unfolding heat capacity change (Δ*C*_p_) in the model (see Eqn 1). Instead, we estimated this parameter by performing DSC experiments in the presence of low concentrations of GdmHCl (Fig. 3).

It is evident that NB‐AGT‐2 is significantly more thermostable that NB‐AGT‐1 and ‐6 (about 15 °C higher *T*_m_) (Fig. 1 and Table 1). This higher thermostability could arise from either higher thermodynamic stability (at all temperatures), a higher temperature for maximal thermodynamic stability or from different temperature‐dependencies of thermodynamic stability (e.g. different unfolding heat capacities, Δ*C*_p_) [28]. Importantly, reversibility tests as well as analysis of DSC transitions using a non‐explicit two‐state unfolding model (Table 1), and the lack of significant scan rate dependence (Figs 2 and 3) supported that DSC analyses using a simple two‐state unfolding model provide physically meaningful information on the unfolding free energy changes (Δ*G*) around the *T*_m_ (please, see the values for the Δ*H*_cal_/Δ*H*_VH_ very close to unity, consistent with a two‐state unfolding behavior) (Table 1). Since the value of the Δ*C*_p_ for each NB‐AGT cannot be extracted from these DSC analyses, we estimated an average of the Δ*C*_p_ = 0.8 ± 0.2 kcal·mol^−1^ from the global *T*_m_‐dependence of Δ*H*_cal_ (Fig. 3A; the *T*_m_ was gradually decreased for each NB‐AGT by addition of low and non‐denaturing GdmHCl concentrations).

### Reversible chemical denaturation at different temperatures

In an attempt to determine the stability curve (Δ*G* vs *T*, Eqn 5) of NB‐AGTs, we combined the DSC analyses (that monitored unfolding at high temperatures) with chemical denaturation analyses at lower temperatures (18–46 °C). The use of GdmHCl as denaturant is justified because it causes reversible unfolding of NB (Fig. S2) and it is a stronger denaturant than urea (the latter does not fully denature some NB) [14].

**Fig. 4:**
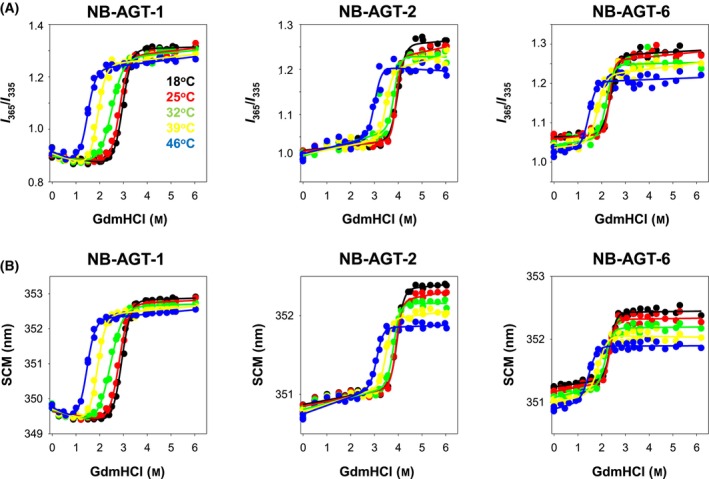
GdmHCl induced isothermal denaturation of NB‐AGT at different temperatures. Different panels show the results from these experiments using different feature of the intrinsic emission fluorescence spectra (A, the *I*_365_/*I*_335_ ratio; B, the SCM) for the indicated NB‐AGT. Best fits using Eqn (4) are shown as lines. The results for different temperatures were shown according to the color code.

**Fig. 5:**
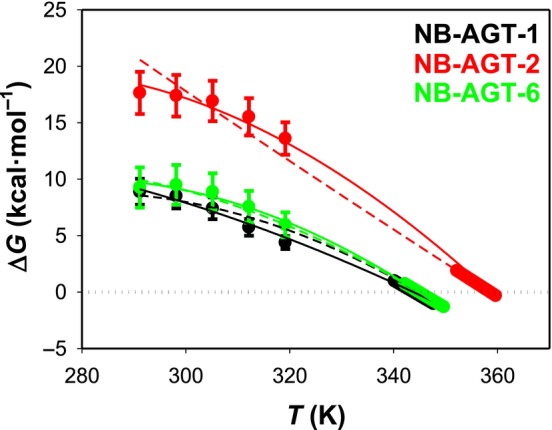
Stability curves built using chemical and thermal denaturation data. Data for high temperature (close to the *T*_m_ value) were obtained from DSC experiments at 2 K·min^−1^ (Fig. 1). Data for GdmHCl denaturation were globally fitted using both fluorescence features (SCM and *I*_365_/*I*_335_) to yield a single Δ*G* value for each variant and temperature (Fig. 4). Data for NB‐AGT‐1, 2 and 6 were successfully fitted to Eqn (5) with Δ*C*_p_ and Δ*H* values as floating parameters (solid lines) or with only Δ*C*_p_ value as floating parameter (dashed lines). Using the former approach, Δ*C*_p_ values (in kcal·mol^−1^·K^−1^) were 0.52 ± 0.12 (NB‐AGT‐1, *r* ^2^ = 0.996), 1.49 ± 0.22 (NB‐AGT‐2, *r* ^2^ = 0.996) and 1.45 ± 0.13 (NB‐AGT‐6, *r* ^2^ = 0.998), whereas Δ*H* values (in kcal·mol^−1^) were 73.4 ± 3.4 (NB‐AGT‐1), 151.3 ± 6.8 (NB‐AGT‐2) and 103.3 ± 3.0 (NB‐AGT‐6). Using the latter approach, Δ*C*_p_ values (in kcal·mol^−1^·K^−1^) were 1.30 ± 0.05 (NB‐AGT‐1, *r* ^2^ = 0.990), 0.03 ± 0.08 (NB‐AGT‐2, *r* ^2^ = 0.985) and 1.07 ± 0.04 (NB‐AGT‐6, *r* ^2^ = 0.997) (in kcal·mol^−1^·K^−1^). The *T*_m_ and/or Δ*H* fixed values are those compiled in Table 1 (*T*_m_ and/or Δ*H*_cal_).

Chemical denaturation was followed by intrinsic tryptophan emission fluorescence, to use low protein concentrations and maximize reversible unfolding. The two spectral features (the ratio of intensities at 365 and 335 nm, *I*_365_/*I*_335_, and the spectral center of mass, SCM) showed similar trends and a single transition, supporting that a two‐state unfolding model is a reasonable description for the chemical denaturation of all NB‐AGT investigated (Fig. 4). Data suggested that the higher thermostability of NB‐AGT‐2 roots in a higher maximal Δ*G* at all temperatures tested (about 8–9 kcal·mol^−1^ at 25 °C, Fig. 5), whereas the temperature‐dependence of the unfolding free energy (related to the unfolding heat capacity) seems to be quite similar. All *m*_eq_ were also similar, with average values (from different temperatures) of 3.1 ± 0.4 (NB‐AGT‐1), 4.4 ± 0.5 (NB‐AGT‐2) and 4.1 ± 0.8 (NB‐AGT‐6) kcal·mol^−1^·M^−1^ supporting that their native and chemically unfolded states are quite similar overall [31]. Actually, the theoretical *m*_eq_ value for proteins of these sizes is 3.3 ± 0.1 kcal·mol^−1^·M^−1^ based on structure‐energetics correlations [31]. When these average values of *m*_eq_ for a given variant were used, we observed that at their maximum, the Δ*G* value was ~ 18 kcal·mol^−1^ for NB‐AGT‐2 and ~ 9–10 kcal·mol^−1^ for NB‐AGT‐1 and 6 (Fig. 5). Estimations of the Δ*C*_p_ could also be determined from stability curves (Fig. 5) with values of 1.5 ± 0.1 kcal·mol^−1^·K^−1^ for NB‐AGT‐2 and NB‐AGT‐6, whereas the estimated Δ*C*_p_ value was 0.5 ± 0.1 kcal·mol^−1^·K^−1^ for NB‐AGT‐1 (Fig. 5). By fixing the *T*_m_ and Δ*H* values to those experimentally obtained (Table 1), a Δ*C*_p_ value of 1.3 ± 0.1 (NB‐AGT‐1) and 1.1 ± 0.1 (NB‐AGT‐6) kcal·mol^−1^·K^−1^ was obtained, but failed to provide a reasonable estimate of the Δ*C*_p_ value (~ 0 kcal·mol^−1^·K^−1^ for NB‐AGT‐2). Even though these analyses support that the data obtained to build the stability curves are not robust enough to obtain accurate Δ*C*_p_ values, they experimentally support the main conclusion of this work: a higher thermodynamic stability at all temperatures and a higher maximal thermodynamic stability (at *T* ~ 20 °C) mainly determine the higher NB‐AGT‐2 thermostability (Fig. 5). It is also worth noting that Δ*C*_p_ values seem somewhat higher than those obtained from DSC experiments in the presence of low GdmHCl concentrations (0.8 ± 0.2 kcal·mol^−1^·K^−1^; Fig. 3A) but still lower than the value predicted from structure‐energetic relationships for proteins of these sizes (1.7–1.8 kcal·mol^−1^·K^−1^) [31, 32]. Overall, the comparison of theoretical and experimental *m*_eq_ and Δ*C*_p_ values for this set of NB‐AGT variants also suggest that the chemically and thermally unfolded state of the NB are structurally different (the former being less structured), in agreement with recent studies using several proteins [33]. Noteworthy, the reversibility of thermal denaturation of all NB‐AGT was enhanced by addition of low concentrations of GdmHCl (up to 80–100% reversibility; Fig. 3B).

### Similar predicted structures for the NB‐AGT

**Fig. 6:**
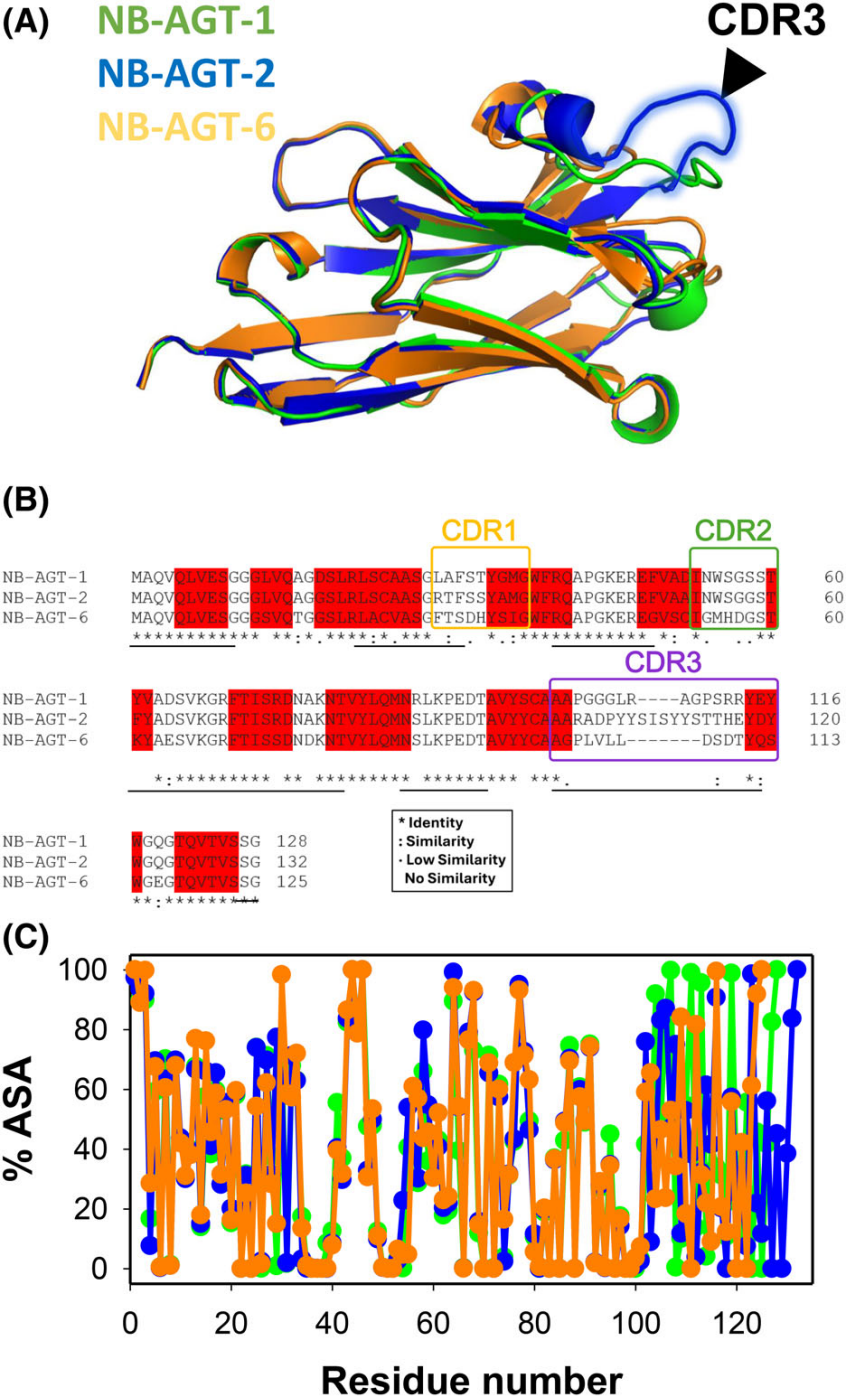
Structural models and sequence alignment for NB‐AGTs. (A) Highest‐scored structural models generated by Alpha‐Fold 2 [29] for NB‐AGT‐1 (in green), NB‐AGT‐2 (in blue) and NB‐AGT‐6 (in orange). (B) Sequence identity for a multiple alignment (using clustalomega; https://www.ebi.ac.uk/Tools/msa/clustalo/) of NB‐AGT‐1, ‐2 and ‐6. The identity or similarity between amino acids is shown using the symbols found in the inset. Amino acids belonging to a β‐sheet structure are highlighted in red, those belonging to CDRs are boxed and the HVL (identified by secondary structural analyses provided by Alpha‐Fold 2) are highlighted in green, all these based on the structural alignment shown in panel A. Plots below the sequence alignment indicate those residues which are buried (using a cut‐off for a buried residue of ≤ 20% of its total ASA using getarea [30]; https://curie.utmb.edu/getarea.html). The *y*‐axis of these plots show the fraction of structural models (from 0 to 1) that shows a buried residue. (C) The accessible surface area (% ASA; average of the three best‐scored models generated by Alpha‐Fold 2 for each NB‐AGT) determined using getarea [30] for NB‐AGT. The color code in panel C is the same that in panel A. Note that in all three panels, the highest divergence is found in the CDR3.

To get an estimation of the structural differences between the three NB‐AGT, we have used the recently developed algorithm Alpha‐Fold 2 [29] and the sequences of the NB‐AGT. A structural alignment of the three NB‐AGT models with the highest rank showed a very similar overall structure (pairwise Cα RMSD of 0.25 Å for NB‐AGT‐1 vs. NB‐AGT‐2 and 0.39 Å vs. NB‐AGT‐6) (Fig. 6A). Visual inspection of this superposition showed a clear difference in the HVL‐3 (belonging to the CDR‐3) (Fig. 6A). A multiple sequence alignment showed high conservation (identity of 60.6–64.0%, including similar residues of 70.5–74.4%), particularly in the β‐sheets, whereas the VHL1‐2 and surroundings (i.e. CDR1‐2) showed a moderate degree of conservation and the VHL‐3 (and CDR3) displayed virtually no conservation (Fig. 6B). Calculation of ASA in the modeled structure using getarea showed very similar solvent exposure of different structural regions, with high exposure of the three CDR (Fig. 6B,C).

Simple structural analyses of the top ranked AlphaFold 2 models help gaining some insight into the relationship between the structure and thermodynamic stability of NB‐AGTs. Notably, NB‐AGT‐2 possesses a longer CDR3 sequence (Fig. 6A,B) compared to NB‐AGT‐1 and NB‐AGT‐6. The CDR3 region often exhibits the highest variability among nanobodies, significantly influencing antigen binding and structural stability [7]. A longer CDR3 in NB‐AGT‐2 may contribute to a greater flexibility, potentially allowing for enhanced transient interactions with surrounding residues, thereby stabilizing the overall structure.

Moreover, the total solvent accessible surface area (SASA) values reveal that NB‐AGT‐2 has the largest SASA (6556.36 Å^2^), followed by NB‐AGT‐1 (6494.84 Å^2^) and NB‐AGT‐6 (6309.68 Å^2^). This higher SASA for NB2 may suggest a higher contribution to structural stability by promoting favorable interaction networks and enhancing surface packing [34, 35]. In addition, the total number of hydrogen bonds supports this hypothesis, as NB‐AGT‐2 forms more hydrogen bonds (93), than NB‐AGT‐1 (84) and NB‐AGT‐6 (86). The higher hydrogen bond count is indicative of increased structural rigidity, which may enhance thermodynamic stability by mitigating the likelihood of local unfolding events [34].

### Binding affinity does not correlate with NB stability

To determine whether thermodynamic stability of NB‐AGT correlates with binding affinity for AGT‐LM, we have determined these affinities by SPR (Fig. S1). All NB‐AGT variants bind tightly to AGT‐LM, even though binding affinities (NB‐AGT‐6 ≫NB‐AGT‐2 > NB‐AGT‐1; Fig. S1) do not show a correlation with thermodynamic stability (NB‐AGT‐2 ≫ NB‐AGT‐1 ~ NB‐AGT‐6; Fig. 5) at 25 °C or with the length of CDR3 (Figs S1 and 6).

In conclusion, our study shows that reversible chemical unfolding at storage or physiological temperatures can be described by equilibrium thermodynamics and correlate with thermal stability for our NB‐AGT, supporting the results of previous studies with other NB [14, 24]. It would be interesting to carry out kinetic denaturation experiments using GdmHCl to know whether unfolding (as an unfolding rate constant *k*_u_) and aggregation (as an aggregation rate constant *k*_agg_) also correlate with thermodynamic stability at storage or physiological temperatures. We must note that these two rate constants may differ by orders of magnitudes at the same temperature [15, 36]. Therefore, we must be cautious when data obtained at very harsh conditions (close to the apparent *T*_m_) are used to compare the thermal stability with the thermodynamic/kinetic stability at lower (storage or physiological) temperatures [17, 18, 19] for a given NB.

## Author contributions

ALP was responsible for conceptualization. AG‐M purified proteins and carried out experiments. MC‐M and ALP carried out data analysis. ESR provided reagents. MC‐M, ESR and ALP. wrote the paper and all authors contributed to editing and revising the manuscript.

## Supporting information


**Fig. S1.** SPR analyses for the NB‐AGT variants interacting with AGT‐LM.
**Fig. S2**. Reversibility of GdmHCl denaturation of NB‐AGT‐1, NB‐AGT‐2 and NB‐AGT‐6.
**Table S1**. Thermodynamic parameters for the thermal unfolding of NB‐AGTs.

**Data S1.** Atomic coordinates for top‐ranked models of NB‐AGT‐1, NB‐AGT‐2 and NB‐AGT‐6 as provided by the Alpha‐Fold 2 algorithm.

## Data Availability

Data available on request from the authors.
