# Supplementary material for: Thermodynamic versus kinetic basis for the high conformational stability of nanobodies for therapeutic applications
Source: FEBS Lett. 2024 Nov 26;599(5):766–76. doi: 10.1002/1873-3468.15064 (PMC11891404; doi:10.1002/1873-3468.15064)
Supplement: Supplementary file 1 — Fig. S1. SPR analyses for the NB‐AGT variants interacting with AGT‐LM. Fig. S2. Reversibility of GdmHCl denaturation of NB‐AGT‐1, NB‐AGT‐2 and NB‐AGT‐6. Table S1. Thermodynamic parameters for the thermal unfolding of NB‐AGTs. [file FEB2-599-766-s001.docx]

**Thermodynamic versus kinetic basis for the high conformational stability of nanobodies for therapeutic applications**

**Atanasio Gómez-Mulas, Mario Cano-Muñoz, Eduardo Salido Ruiz and Angel Luis Pey.**

**Supplementary Information**

**Figure S1. SPR analyses for the NB-AGT variants interacting with AGT-LM.** Blank subtracted SPR sensograms corresponding to a Single Cycle Kinetics experiment performed with different NB-AGTs immobilized in the chip and AGT-LM as analyte. Five AGT-LM concentrations were sequentially injected during 120 s with 30 s stabilization (dotted vertical lines). Data from two replicas were globally analysed using Biacore T200 Evaluation Software to yield the *K*_d_ values.

**Figure S2. Reversibility of GdmHCl denaturation of NB-AGT-1 (A), NB-AGT-2 (B) and NB-AGT-6 (C).** Left and right panels show tryptophan fluorescence emission spectra at 18^o^C or 46^o^C, respectively. Samples contained: no denaturant (black lines. 5 µM protein), high concentration of denaturant at the indicated temperature and denaturant concentration as described in the main text (red lines, 5 µM protein) and refolding conditions at which a similar sample with denaturant was diluted 1:1 in buffer (10 µM protein and diluted to 5 µM protein and half of the initial denaturant concentration) and incubated for 2 h at 18^o^C or 46^o^C (blue lines). Denaturant concentrations were selected to provide conditions for reversible refolding (from red to blue and black lines)

**Table S1. Thermodynamic parameters for the thermal unfolding of NB-AGTs.** Values are the mean ± s.d. from one experiment at three scan rates (1 K·min^-1^, 2 K·min^-1^ and 4 K·min^-1^)(see Figure 2 of the main text).

| **Variant** | **Thermodynamic parameter** | |
| --- | --- | --- |
|  | ***T*_m_ (K)** | **Δ*H*_VH_ (kcal·mol^-1^)** |
| **NB-AGT-1** | 344.16 ± 0.15 | 93.7 ± 1.0 |
| **NB-AGT-2** | 358.56 ± 0.08 | 115.6 ± 3.7 |
| **NB-AGT-6** | 344.71 ± 0.34 | 88.2 ± 1.7 |
